# Acetylation-modulated communication between the H3 N-terminal tail domain and the intrinsically disordered H1 C-terminal domain

**DOI:** 10.1093/nar/gkaa949

**Published:** 2020-10-30

**Authors:** Fanfan Hao, Kevin J Murphy, Tomoya Kujirai, Naoki Kamo, Junko Kato, Masako Koyama, Akimitsu Okamato, Gosuke Hayashi, Hitoshi Kurumizaka, Jeffrey J Hayes

**Affiliations:** Department of Biochemistry and Biophysics, University of Rochester Medical Center, Rochester, NY 14642, USA; Department of Biochemistry and Biophysics, University of Rochester Medical Center, Rochester, NY 14642, USA; Laboratory of Chromatin Structure and Function, Institute for Quantitative Biosciences, The University of Tokyo 1-1-1 Yayoi, Bunkyo-ku, Tokyo 113-0032, Japan; Department of Chemistry and Biotechnology, Graduate School of Engineering, The University of Tokyo, 7-3-1 Hongo, Bunkyo-ku, Tokyo 113–8656, Japan; Laboratory of Chromatin Structure and Function, Institute for Quantitative Biosciences, The University of Tokyo 1-1-1 Yayoi, Bunkyo-ku, Tokyo 113-0032, Japan; Laboratory of Chromatin Structure and Function, Institute for Quantitative Biosciences, The University of Tokyo 1-1-1 Yayoi, Bunkyo-ku, Tokyo 113-0032, Japan; Department of Chemistry and Biotechnology, Graduate School of Engineering, The University of Tokyo, 7-3-1 Hongo, Bunkyo-ku, Tokyo 113–8656, Japan; Research Center for Advanced Science and Technology, The University of Tokyo, 4-6-1 Komaba, Meguro-ku, Tokyo 153–8904, Japan; Department of Biomolecular Engineering, Graduate School of Engineering, Nagoya University, Furo-cho, Chikusa-ku Nagoya 464-8603, Japan; Laboratory of Chromatin Structure and Function, Institute for Quantitative Biosciences, The University of Tokyo 1-1-1 Yayoi, Bunkyo-ku, Tokyo 113-0032, Japan; Department of Biochemistry and Biophysics, University of Rochester Medical Center, Rochester, NY 14642, USA

## Abstract

Linker histones (H1s) are key structural components of the chromatin of higher eukaryotes. However, the mechanisms by which the intrinsically disordered linker histone carboxy-terminal domain (H1 CTD) influences chromatin structure and gene regulation remain unclear. We previously demonstrated that the CTD of H1.0 undergoes a significant condensation (reduction of end-to-end distance) upon binding to nucleosomes, consistent with a transition to an ordered structure or ensemble of structures. Here, we show that deletion of the H3 N-terminal tail or the installation of acetylation mimics or *bona fide* acetylation within H3 N-terminal tail alters the condensation of the nucleosome-bound H1 CTD. Additionally, we present evidence that the H3 N-tail influences H1 CTD condensation through direct protein-protein interaction, rather than alterations in linker DNA trajectory. These results support an emerging hypothesis wherein the H1 CTD serves as a nexus for signaling in the nucleosome.

## INTRODUCTION

The eukaryotic genome is packaged into nucleosomes, which consists of a nucleosome core, linker DNA, and linker histone (H1). The nucleosome core is comprised of 147 bp DNA segments wound around the core histone octamer, which consists of two copies of each of the core histones H2A, H2B, H3 and H4 (1). Adjacent NCPs are connected by variable length of linker DNA (10–90 bp) to form oligonucleosome arrays (2). H1s bind to the surface of nucleosomes near the dyad and bring the two linker DNA closer together to form a stem-like structure (2,3,4). The H1-induced closure of linker DNA is further supported by a recent cryogenic electron microscopy (cryo-EM) and crystal structures of a 197-bp nucleosome containing a full-length H1, in which the globular domain of H1 binds to the DNA at the nucleosome dyad and interacts with both linker DNA segments (4). The decreased entry-exit angle of nucleosomal linker DNA resulting from the stem-like structure might contribute to a unique zigzag folding pattern of nucleosomes within oligonucleosome arrays that further facilitates chromatin compaction (5). Interestingly, structural transitions in H1 may contribute to distinct nucleosome packing densities and conformations of oligonucleosomes (4,6,7)

Chromatin is a highly dynamic entity and genomic DNA accessibility can be regulated by various mechanisms, including posttranslational modifications (PTMs) on the histone tail domains, ATP-dependent chromatin remodeling, and replacement of canonical histones with specialized variants (8–12). Histone acetylation is generally associated with active transcription, and can directly disrupt nucleosome-nucleosome interactions or histone-DNA contacts as well as indirectly functioning as a platform that is recognized by various proteins (13–16). Conversely, H1s stabilize the folding of extended nucleosome arrays into higher-order chromatin structures and are depleted at the transcription start site of active genes (5,17–22). However, how histone acetylation regulates chromatin structure in conjunction with the effects of linker histone is still an open question.

In higher eukaryotes, H1s have a tripartite structure composed of a short N-terminal domain (NTD), a conserved trypsin-resistant central globular domain, responsible for structure-specific binding to the nucleosome, and a long, extremely basic ∼100 amino acid C-terminal domain (CTD), which is required for high affinity chromatin binding *in vivo* and is vital for chromatin condensation (23–25). While the globular domain is highly conserved among somatic H1 isoforms (80–100% similarity among human H1s), the CTD is less well conserved (40–80% similarity). Nevertheless, H1 CTDs do exhibit high similarity in overall amino acid composition with ∼40% of somatic H1 CTDs comprised of basic amino acids, nearly all lysines with an occasional arginine, and with alanine, serine and proline comprising most of the remaining residues (2,26,27). H1 CTDs are almost completely devoid of acidic and aromatic residues. These sequence features are characteristic of intrinsically disordered proteins (IDPs) (27). Although the H1 CTD is unstructured in aqueous solution, peptides derived from this domain exhibit secondary structures when bound with DNA or in secondary structure stabilizing solvents such as TFE (28,29). Recent work employing Förster Resonance Energy Transfer (FRET) reveals that the H1 CTD condenses upon interaction with nucleosomes, consistent with a disordered to ordered transition (25,30). However, whether the condensed H1 CTD adopts defined structure or an ensemble of structures is unclear. In addition, the H1 CTD is less condensed when associated with oligonucleosome arrays compared to that induced when bound to mononucleosomes (6). Importantly, H1 CTD structure appears to be tightly coupled to linker DNA trajectory, which is altered by the presence of neighboring nucleosomes in arrays. The coupling of CTD structure and the conformation of linker DNA within the nucleosome array raises the possibility of an additional level of regulation of chromatin structure wherein altering the propensity for changes in CTD condensation may tune the accessibility within condensed chromatin (6).

Core histone tails protrude outside the nucleosome core particle, either through the channels formed by aligned superhelical gyres of DNA (H2B N-terminal tail and H3 N-terminal tail) or over/under them (H2A N-terminal tail and H4 N-terminal tail) (31). Although not essential for mononucleosome structure, the core histone tails are required for higher-order chromatin structure formation (32). Removal of the H4 N-terminal tail reduces MgCl_2_-dependent folding and self-association of reconstituted nucleosome arrays (33). Acetylation of H4K16 (Lys residue within the H4 N-terminal tail) also impairs the ability of oligonucleosomes to undergo salt-dependent compaction (34). The H3 N-terminal tail switches from intra-nucleosome interactions in extended oligonucleosome arrays to inter-nucleosome interactions in condensed chromatin (35).

The H3 N-terminal tail domains project out of the nucleosome, between DNA superhelical gyres, about one helical turn to either side of the center of the nucleosome, near the canonical H1 binding site on the nucleosome surface (31). Therefore the H3 tail domains are located near where the linker DNA enters and exits the nucleosome, and crosslinking studies show that they interact with linker DNA in mononucleosomes (36–38). Thus, both H1 and the H3 N-terminal tail interact with the linker DNA. The H1 CTD also modulates H3 N-terminal tail dynamics in nucleosomes and impedes post-translational modifications (PTMs) such as acetylation, methylation and phosphorylation within the H3 N-terminal tail *in vitro* (39). Given the close proximity of the H3 tail domain and the H1 CTD, we reasoned that the H3 tail domain may interact with or otherwise contribute to the binding environment of H1, and therefore might impact the condensation of the H1 CTD (25,30). We therefore investigated whether the H3 tail domain influences the nucleosome-bound structure of the H1 CTD. We found that deletion of the H3 N-terminal tail or the installation of acetylation mimics within H3 N-terminal tail alters the condensation of the H1 CTD, compared to unmodified mononucleosomes. Furthermore, acetylation mimics and bona fide lysine acetylation of the H3 tail domain have identical effects on H1 CTD condensation. Interestingly, analysis of H3 tail modifications on linker DNA trajectory and H3 tail crosslinking studies indicate that the H3 tail communicates directly with the H1 CTD.

## MATERIALS AND METHODS

### Expression and purification of linker histones and core histones

Linker histone H1(0) from *Xenopus laevis* (here referred to as H1) and the double cysteine mutant (H1G101CK195C) were expressed in bacterial cells BL21(DE3) using the plasmid pET3aH1(0)a (25). Briefly, the coding sequence of H1 was inserted into pET3a vector (Novagen) and linker histones were purified by ion-exchange chromatography using Biorex-70 resin (Bio-Rad) as described (25). H1 concentration was determined by quantitative comparison with an H1 standard, whose concentration had been determined by amino acid analysis (25).

All histone H3 (including H3 tail mutants) used for this study contained a cysteine to alanine substitution at position 110. Histone H3 and H4 were expressed in bacterial cells BL21(DE3). Expression and purification of H3 and H4 was performed as described previously (40), except for the H4 N tail deletion mutant, which was cultured at 30°C. Purified H3/H4 tetramer concentration was determined by quantitative comparison with standard H3/H4 tetramer.

Histone H2A and H2B, including the tail deletion mutants, were expressed in bacterial cells BL21(DE3). Expression and purification of H2A/H2B dimer was performed as described before (41). H2A/H2B dimer concentration was determined by quantitative comparison with standard tetramer.

### Native chemical ligation of acetylated peptide to histone H3

Bona fide acetylated histone H3 proteins were synthesized through one-pot native chemical ligation and desulfurization with methyl thioglycolate (MTG) (42). In brief, chemically synthesized histone H3 N-terminal peptide (1–28) containing specific histone acetylation patterns were ligated with the globular part of histone H3 (A29C-135, C110A). Chemically synthesized N-terminal H3 peptide (470 nmol, 1.90 mg in the case of H3–3Ac2) and expressed C-terminal peptide (162 nmol, 2.45 mg) were dissolved in 62.1 μl of NCL buffer (6 M GdnHCl, 0.2 M NaH_2_PO_4_ at pH 7.0). To the reaction mixture were added 16.2 μl of MTG solution (500 mM in NCL buffer) and 2.7 μl of TCEP solution (600 mM in NCL buffer). The pH was adjusted around 7.0 by the addition of 1 N NaOH aq. The whole mixture was stirred at 37°C for 3 h under argon atmosphere. Then, 54.0 μl of TCEP solution (500 mM), 13.0 μl of glutathione solution (1 M), and 12.2 μl of VA-044 aq. were added, and the reaction solution was stirred at 37°C for 1 h under argon atmosphere. Finally, the whole reaction solution was diluted by 25% acetonitrile aqueous solution, then purified by high performance liquid chromatography (HPLC) to afford the desired ligated and desulfurized products: 2.45 mg (44% yield) for H3-WT, 1.35 mg (37% yield) for H3–6Ac, 0.74 mg (44% yield) for H3–3Ac1 and 1.39 mg (45% yield) for H3–3Ac2. Because cysteine at position 29 was converted to alanine by desulfurization after ligation, no sequence mutation was introduced except for the C110A. Full length H3 bearing specific histone acetylation patterns was identified by MALDI-TOF MS.

### 601 DNA fragments for nucleosome reconstitution

DNA fragments for nucleosome reconstitution were generated by digestion of plasmid p207–12 with EcoRV and isolation of the 207bp DNA fragments, which containing the 601 positioning sequence (43), on 0.8% agarose gels via electro-elution. Labeled 207bp DNA was prepared by PCR using labeled primers (GenScript). Cy3 and Cy5 were conjugated to amino-modifier C6-dT for internal labeling. Forward primer: ATCGGACCC/iCy5N/ATACGCGGCC, reverse primer: AGTAG/iCy3N/ATTAATTAATATGAATTCGGATCCACATGCAC. The position of Cy3 and Cy5 within the primer was indicated by iCy3N and iCy5N, respectively. After PCR, fluorescently labeled 207bp DNA fragments was purified by PCR purification kit (Qiagen).

### Nucleosome reconstitution

Nucleosomes were reconstituted via standard stepwise salt dialysis. Briefly, 10 μg of H3/H4 tetramer, 11.6 μg H2A/H2B dimer and 20 μg 601 DNA was added into reconstitution buffer (10 mM Tris, pH 8.0, 1 mM EDTA, 5 mM DTT and 2 M NaCl) in a total volume of 600 μL, transferred to dialysis tubing then dialyzed against TE containing decreasing concentrations of NaCl at 1.2, 1, 0.8, 0.6M, each for 2 h each at 4°C, followed by dialysis against TE overnight at 4°C.

Reconstituted nucleosomes were purified over 10 ml 7–20% sucrose gradients by ultracentrifugation in a Beckmann SW41 rotor for 18 h at 34 000 g at 4°C. The fractions containing nucleosomes were combined together and concentrated to a final concentration of 0.1–0.3 μM using a microfuge tube filtration unit (EMD Millipore) with a 50 kDa membrane Nominal Molecular Weight Limit. Purified nucleosomes were analyzed on a 0.7% agarose gels and 18% SDS-PAGE.

### Attachment of maleimide-Cy3 and maleimide-Cy5 to linker histone

H1 G101C K195C, in which cysteines were located at either end of the H1 CTD (25), was incubated in 50 mM DTT for one hour to fully reduce cysteine residues, then DTT was removed by Bio-Rex 70 chromatography and the reduced protein solution was immediately frozen on dry ice. Fractions containing reduced H1 G101C K195C were treated with 5–10-fold excess of either maleimide-Cy3, or maleimide-Cy5, or a 50/50 mix of both for 30 min at room temperature in the dark according to manufacturer's instructions (GE Healthcare). The free dyes were removed by another round of Bio-Rex chromatography. The concentration of fluorophore-labeled H1 was determined by quantitative comparison with an H1 standard similarly as above.

### FRET analysis

Fluorophore-labeled H1 (final concentration 5–15 nM) in H1 binding buffer (10 mM Tris–HCl pH 8.0, 50 mM NaCl, 0.3% BSA) was mixed with a range of amounts of purified mononucleosomes as indicated in the figure legends to ensure saturated H1 binding by nucleosomes. Emission spectra were recorded with excitation at 515 nm (Cy3 donor) and 610 nm (Cy5 acceptor) wavelengths with 5-nm slit widths in both excitation and emission channels on a Horiba Jobin Yvon FluoroMax-4 spectrofluorometer. The spectra of H1 binding buffer was also recorded and used for background subtraction. The ratio_(A)_ method was used to determine FRET efficiency as described (44). The value (ratio)_A_ is the emission of acceptor excited at the donor excitation wavelength divided by the emission of acceptor under direct excitation (Equation 1)

(1) $$\begin{equation*}{\left( {{\rm ratio}} \right)_A} = \frac{{E{{\rm{\varepsilon }}^{\rm{D}}}\left( {{\rm{\lambda ^{\prime\prime}}}} \right){{\rm{d}}^ + } + {{\rm{\varepsilon }}^{\rm{A}}}\left( {{\rm{\lambda ^{\prime}}}} \right)}}{{{{\rm{\varepsilon }}^{\rm{A}}}\left( {{\rm{\lambda ^{\prime\prime}}}} \right)}}\end{equation*}$$

(2) $$\begin{equation*}E = \frac{{{{\rm{\varepsilon }}^{\rm{A}}}\left( {{\rm{\lambda ^{\prime\prime}}}} \right){{\left( {{\rm ratio}} \right)}_A} - {{\rm{\varepsilon }}^{\rm{A}}}\left( {{\rm{\lambda ^{\prime}}}} \right)}}{{{{\rm{\varepsilon }}^{\rm{D}}}\left( {{\rm{\lambda ^{\prime}}}} \right){d^ + }}}\end{equation*}$$

E is the FRET efficiency, ϵ^D^ (λ′) and ϵ^A^ (λ′) are the extinction coefficients of donor (Cy3) and acceptor (Cy5), respectively. d^+^ is the fraction of donor labeled molecules, λ′ is the wavelength for Cy3 excitation 515 nm, λ″ is the wavelength for Cy5 excitation at 610 nm. Numerator represents the FRET intensity and denominator represents the fluorescence signal from directly excited acceptor (Equation 1). Note that (ratio)_A_ is independent of acceptor concentration. For this work, ϵ^D^(515) = 92 058 cm^−1^M^−1^ (Cy3), ϵ^A^(515) = 6078 cm^−1^M^−1^ (Cy5), ϵ^A^(610) = 161 103 cm^−1^M^−1^ (Cy5).

To eliminate issues with determination of d^+^ and absolute FRET efficiencies, herein we report the FRET efficiency difference between H1 bound to nucleosome and H1 alone, ΔE (Equation 3) which can be derived from Equations (1) and (2):

(3) $$\begin{equation*}\frac{{\Delta E}}{{{E_{{\rm H1alone}}}}} = \frac{{{{\left( {{\rm ratio}} \right)}_{{\rm Aexp}}} - {{\left( {{\rm ratio}} \right)}_{{\rm AH1alone}}}}}{{{{\left( {{\rm ratio}} \right)}_{{\rm AH1alone}}} - {{\rm{\varepsilon }}^{\rm{A}}}\left( {{\rm{\lambda ^{\prime}}}} \right)/{{\rm{\varepsilon }}^{\rm{A}}}\left( {{\rm{\lambda ^{\prime\prime}}}} \right)}}\end{equation*}$$

ΔE is independent of d^+^ and only one accurate E_H1alone_ is required for the calculation of Δ*E*. Here, we determined d^+^ = 0.66 for labeled H1 alone as described before (44). All determinations are based on *N* ≥ 3 replicates.

The linker DNA end-to-end distance FRET experiment was performed similar to the above H1 CTD FRET experiment. Note that fluorescently labeled nucleosome has Cy3 and Cy5 specifically incorporated at defined DNA ends, so d^+^ = 1. For this work, ϵ^D^(515) = 53160 cm^−1^M^−1^ (Cy3), ϵ^A^(515) = 3749 cm^−1^M^−1^ (Cy5), ϵ^A^(610) = 118 400 cm^−1^M^−1^ (Cy5).

### Electrophoretic mobility shift assay (EMSA)

The 207 bp 601 nucleosomes (70 ng, ∼15 nM) and H1 in amounts stated in the figure legend were incubated at 25°C for 30 min in 20 μl of binding buffer (10 mM Tris–HCl, pH 8.0, 1 mM EDTA, 50 mM NaCl, 150 ng/μl BSA, and 5% (v/v) glycerol). Then reaction mixtures were loaded directly onto a native 5% polyacrylamide gel (acrylamide:bisacrylamide = 19:1) and run at 160 V for 3 h at 4°C. DNA and nucleosome bands were detected by either ethidium bromide staining or on Typhoon imager using Cy5 excitation and emission setting.

### H3 crosslinking

For H1–H3 crosslinking, 4-azido phenacylbromide (APB) modified H3T6C nucleosomes or WT nucleosomes were incubated with Cy5 labeled H1 G101C in binding buffer (10 mM Tris–HCl, pH 8.0, 1 mM EDTA, 50 mM NaCl and 5% v/v glycerol) for 30 min at 25°C. The reactions were placed in a pyrex tube and irradiated at 365 nm for 90 s as previously described (4,37). The H1/H3 crosslinking products were analyzed by separation of components on 15% SDS-PAGE gel and imaging of Cy5 labeled constituents on a Typhoon imager using Cy5 excitation and emission filters. H3 tail crosslink mapping to DNA was carried out as described (37). Briefly, nucleosomes were reconstituted with APB-modified H3 T6C and 207 601 DNA fragments radiolabeled at the 5′ end of one of the two strands. Nucleosomes were incubated for 10 min. in 10 mM Tris, pH 8.0, 1 mM EDTA, 50 mM NaCl, in the absence or presence of H1 and crosslinking was initiated as described above. The DNA was isolated from irradiated samples, strand breaks generated at sites of crosslinking, and sites of crosslinks mapped by running products on 6% polyacrylamide denaturing (sequencing) gels as described (37).

## RESULTS

### Deletion of the H3 N-terminal tail domain alters H1 CTD condensation

**Figure 1.**
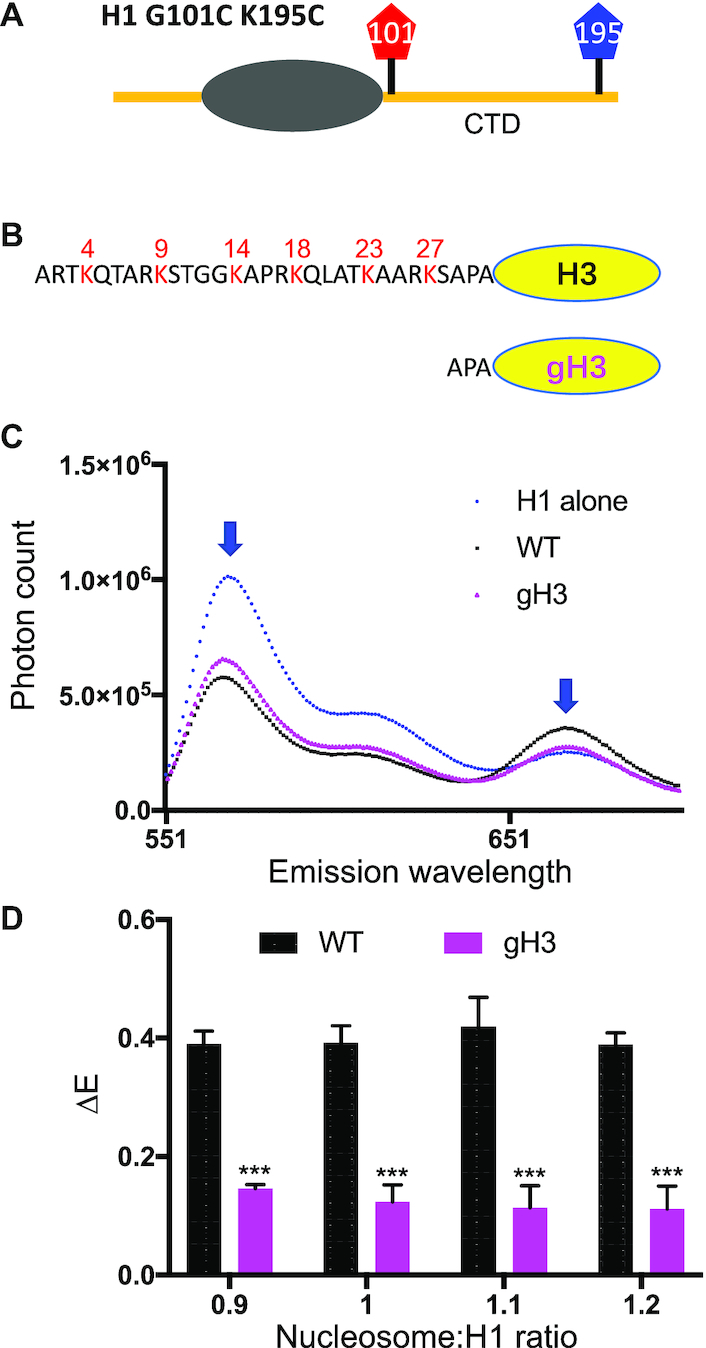
The H3 tail domain influences H1 CTD structure in the nucleosome. Nucleosomes were reconstituted with either full-length H3 (WT) or H3 lacking the N-terminal tail domain (gH3) and FRET assays performed with H1 labeled with Cy3/Cy5 at either end of the H1 CTD. (**A**) H1 G1010C K195C was labeled with the fluorophores Cy3 and Cy5. (**B**) Schematic showing WT H3 and deletion of the N-terminal 28 residues in gH3. (**C**) Emission spectra for H1 alone (blue), and H1 bound to WT nucleosomes (black) or gH3 nucleosomes (magenta). Cy3 excitation was at 515 nm; the Cy3 and Cy5 emission peaks (∼560 and ∼670 nm) are indicated by blue arrowheads. (**D**) Plot of FRET efficiency difference (Δ*E*) for samples with the indicated nucleosome:H1 ratios. Δ*E* was calculated as the difference in FRET efficiency for H1-nucleosome complexes and free H1 for independent trials. Error bars reported are standard deviations (SDs). P values represent probabilities associated with two-tailed Student's *t*-test. *N* ≥ 3. (***), *P* < 0.001.

Given the close proximity of the H3 tail domains and the H1 binding site in the nucleosome and the effect of H1 CTD on enzymatic modification of H3 tail (36,39), we hypothesized that the H3 N-terminal tail may influence the environment and thus the structure of the nucleosome-bound H1 CTD. To test this hypothesis, we reconstituted nucleosomes with recombinant core histones containing either full length H3 (WT) or H3 lacking the N-terminal tail domain (gH3), and employed a FRET approach to monitor the extent of condensation of the H1 CTD upon binding to WT or gH3 nucleosomes (Figure 1A and B, Supplementary Figure S1). We measured the difference in FRET efficiency (Δ*E*) between nucleosome bound and free labeled H1 over a range of nucleosome:H1 concentrations to ensure saturated (1:1) nucleosome binding by H1. Consistent with the previous work, acceptor Cy5 fluorescence emission (peak ∼ 670 nm) exhibited an increase upon binding of labeled H1 to WT nucleosomes and a resultant increase in FRET (Δ*E*), indicating a reduction in the end-to-end distance across the H1 CTD upon binding to nucleosomes (Figure 1C and D). We refer to the nucleosome-dependent change in the H1 CTD as ‘condensation’. Importantly, deletion of the H3 N-terminal tail domain resulted in a significant reduction in the FRET response compared to WT nucleosomes (Figure 1C). Quantification showed that H1 binding to WT nucleosomes exhibited a Δ*E* of ∼0.4 which decreased to ∼0.18 for H1 binding to gH3 nucleosomes (Figure 1D). Note in both cases Δ*E* reaches constant values at nucleosome:H1 ratios approximately ≥1, indicating saturation of H1 binding to nucleosomes. Importantly, nucleosome-dependent H1 CTD was unaffected by deletion of either the H2B or H4 tail domains (Supplementary Figure S2A and B). These results show that the H3 N-terminal tail domain—but not those of H2B or H4—is required for full condensation of the H1 CTD in nucleosomes.

To investigate whether the H3 N-terminal tail domain affects H1 CTD condensation in chromatin complexes beyond mononucleosomes, we extended our analysis of the extent of H1 CTD condensation to asymmetric dinucleosomes. These dinucleosomes contain a 207 bp nucleosome spacing (30-*N*-60-*N*-0, where *N* = 147 bp ‘core’ DNA), with a 30 bp linker DNA on one side of the dinucleosome (Supplementary Figure S4A and B). We chose the asymmetric dinucleosome as we have shown that the extent of condensation of the H1 CTD is identical to that of longer nucleosome arrays (6). Moreover, H1 binds preferentially to the nucleosome within the asymmetric dinucleosome that has two linker DNA segments (Supplementary Figure S4A and B), which avoids complications due to intermolecular (H1–H1) FRET (6). Consistent with previous findings, the H1 CTD undergoes condensation upon binding to the dinucleosome, but is ultimately somewhat less compact compared to H1 associated with mononucleosomes (Supplementary Figure S4C) (6). Importantly, H1 binding to dinucleosomes containing gH3 results in a significant decrease in H1 CTD condensation compared to dinucleosomes containing WT H3, consistent with results observed in mononucleosomes (Supplementary Figure S4C).

**Figure 2.**
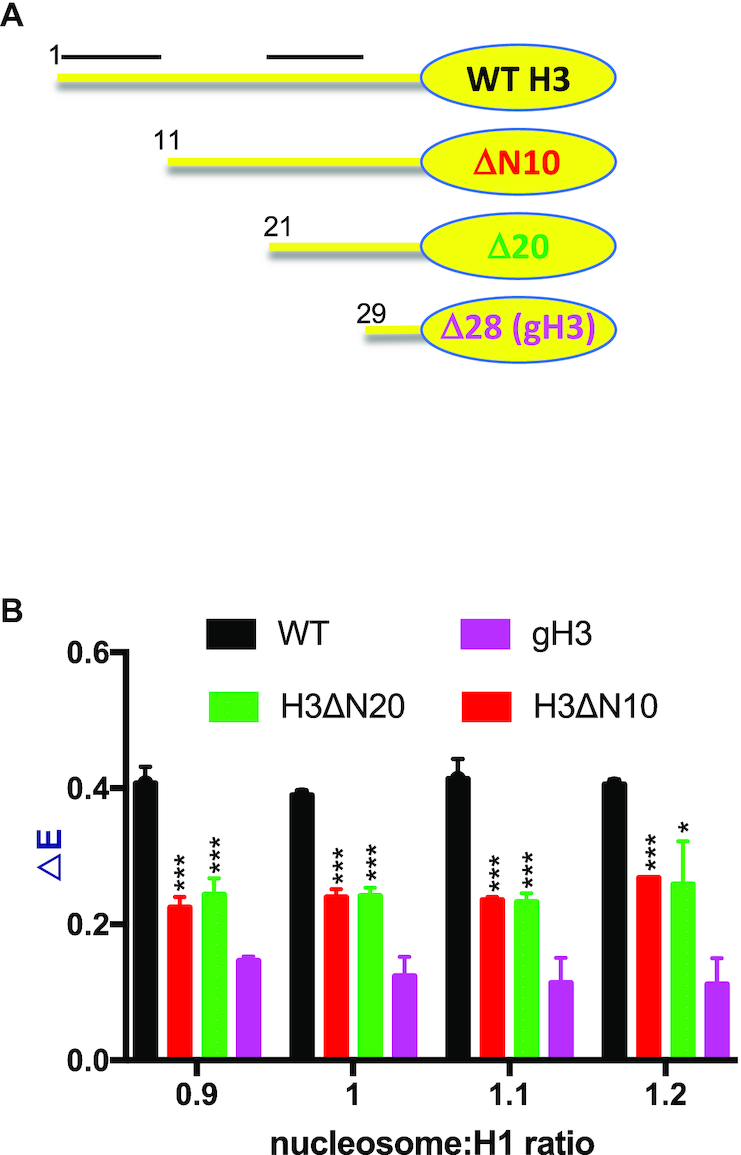
Two regions within the H3 tail affect H1 CTD condensation in nucleosomes. (**A**) Schematic of WT H3 and H3 ΔN10, ΔN20 and gH3 with N-terminal tail deletions of 10, 20 and 28 residues, respectively. (**B**) FRET response (ΔE) for WT H3 (black), H3 ΔN10 (red), H3 ΔN20 (green) and gH3 (magenta) assessed at the nucleosome:H1 ratios indicated below the graph. Numbers in (A) indicate the first residue in each protein. Bars above the schematic for WT H3 indicate regions that affect H1 CTD structure. (***) *P* < 0.001, (*) *P* < 0.05.

To determine whether a distinct sub-region of the H3 tail is required for H1 CTD condensation we prepared two additional H3 mutants with partial tail deletions (Figure 2A). We found that deletion of the first ten residues of the H3 tail (Δ10) reduced H1 CTD condensation as detected by our FRET assay, while deletion of an additional ten residues (Δ20) did not result in additional change in overall H1 CTD structure (Figure 2B). However, deletion of residues 21–28 to generate gH3 results in a further reduction in H1 CTD condensation. These results suggest that two distinct regions within the H3 tail contribute to defining the structure of nucleosome-bound H1.

### H3 N-tail acetylation mimics reduce H1 CTD condensation

**Figure 3.**
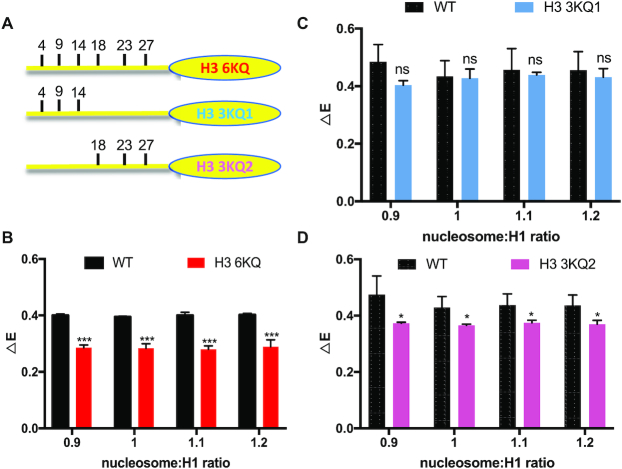
Mimics of lysine acetylation in the H3 tail domain affect H1 CTD condensation. H3 proteins containing K → Q substitutions at known sites of lysine acetylation were incorporated into nucleosomes and the extent of H1 CTD condensation determined by FRET analysis. (**A**) Schematic showing sites of K → Q substitution in the H3 tail domain. (**B–D**) FRET analysis of labeled H1 bound to H3 6KQ, H3 3KQ1 and H3 3KQ2 nucleosomes compared to WT at the nucleosome: H1 ratios indicated in the figure legend. NS, no significant difference; (***) *P* < 0.001, (*) *P* < 0.05.

We next set out to determine whether other modifications of the H3 N-terminal tail domain influence H1 CTD structure. Six lysine residues within H3 tail (K4, K9, K14, K18, K23 and K27) have been identified as sites of acetylation *in vivo*. We changed these six lysines to glutamine to mimic acetylated lysine (H3 6KQ) and prepared nucleosomes (Figure 3A). Titrations of H3 6KQ nucleosomes with the labeled H1 resulted in a Δ*E* of ∼0.28, significantly less than that observed for H1 binding to the WT nucleosome (Figure 3B). Thus, similar to H3 tail deletions, the acetylation mimics affected the extent of condensation of the nucleosome-bound H1 CTD. To further determine which K→Q substitutions within the H3 tail are responsible for the reduction in H1 CTD condensation, we constructed mutants H3 3KQ1, and H3 KQ2, in which lysines K4, K9 and K14 or lysines K18, K23 and K27 within the H3 N-terminal tail domain were replaced by glutamines, respectively (Figure 3A). We observed that while ΔE was not significantly changed by inclusion of H3 3KQ1 in nucleosomes, ΔE, and thus the extent of H1 CTD condensation was significantly reduced upon interaction with H3 3KQ2 nucleosomes compared to WT nucleosomes (Figure 3C and D), Thus lysine acetylation mimics installed at K18, K23 and K27 in the more interior region of the H3 N-terminal tail, but not those near the N-terminus, affect H1 CTD condensation. Importantly, no single acetylation mimic installed at any of the interior three positions resulted in a change in FRET efficiency, nor did replacing H3 serine 10 with glutamic acid to mimic S10 phosphorylation (Supplementary Figure S2C and D). We also extended the analysis to investigate acetylation mimics within the H4 N-terminal tail. In accord with the lack of effect of H4 tail removal, acetylation mimics within H4 N-tail at K5, K8, K12 and K16 did not affect H1 CTD condensation (data not shown). In total, these results indicate that mimics of lysine acetylation present at specific locations within the H3 tail domain, but not H3 S10 phosphorylation or modifications of other N-terminal tail domains, alters the structure of nucleosome-bound H1.

### H3 N-terminal tail modification does not affect H1 CTD condensation through changes in linker DNA trajectory

**Figure 4.**
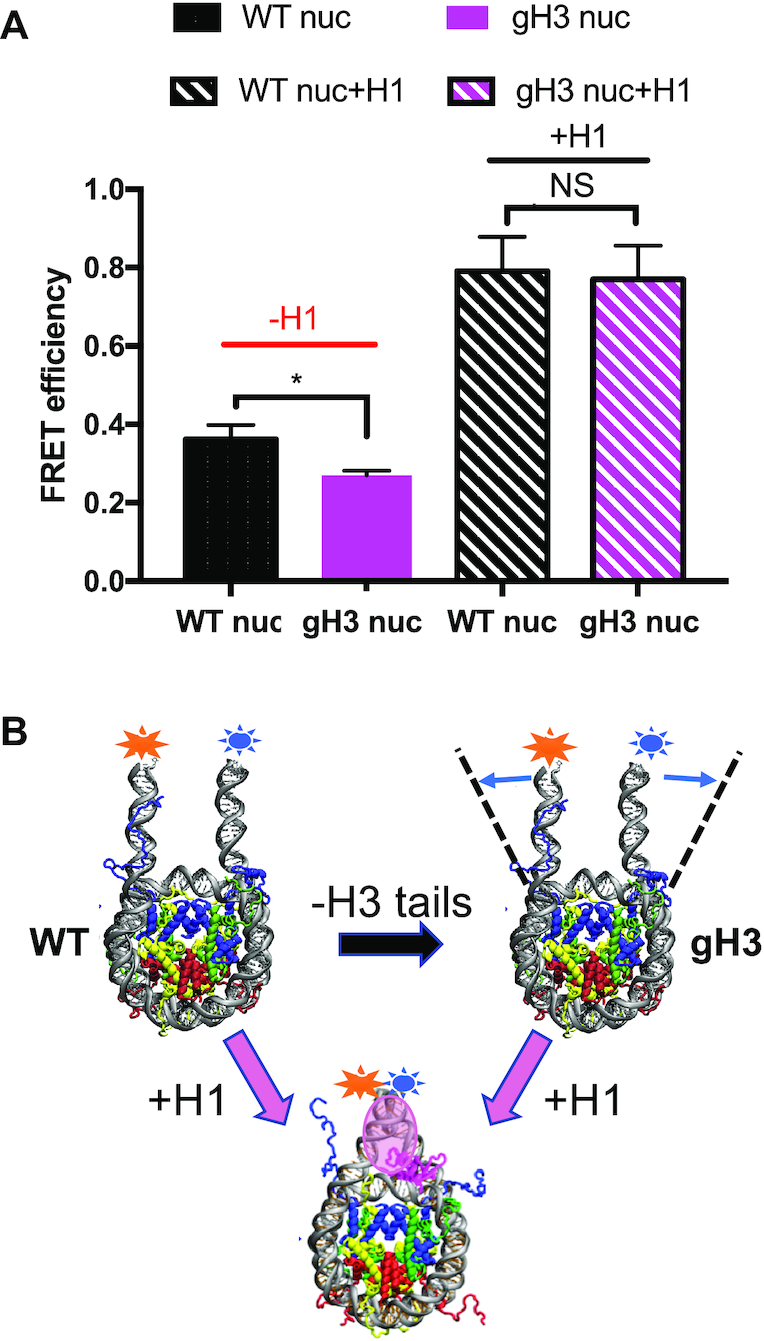
Linker DNA end-to-end distance is similar in H1-bound WT and gH3 nucleosomes. (**A**) FRET efficiency was determined for WT and gH3 nucleosomes containing Cy3 and Cy5 labels attached near the ends of the DNA (see Materials and Methods) in the absence or presence of H1, as indicated. (*), *P* < 0.05; NS, no significant difference. (**B**) Schematic describing changes in linker DNA end-to-end distance observed in (A).

Previous work has shown that the extent of H1 CTD condensation is dependent on linker DNA trajectory (6). To determine whether the H3 N-terminal tail modifications investigated above alter H1 CTD condensation via changes in linker DNA path, we reconstituted nucleosomes in which the DNA ends were labeled with FRET donor and acceptor fluorophores. We then monitored FRET to determine whether H3 modifications altered linker DNA trajectory in different nucleosomal contexts. We observed a substantial loss in FRET between linker DNA ends in gH3 nucleosomes compared to WT, indicated an opening of nucleosome linker DNA upon removal of the H3 N-terminal tail, consistent with prior observations (45). The calculated average linker DNA end-to-end distance for the WT nucleosome is ∼6.04 nm, which increased to ∼6.63 nm for the gH3 nucleosome (Figure 4A and B). The variation of linker DNA geometry is further confirmed by electrophoretic mobility shift assay (EMSA), as gH3 nucleosomes migrate more slowly through the gel compared to WT nucleosomes, consistent with a more open nucleosome conformation (Supplementary Figure S3). In agreement with cryo-EM structure analysis (4,5) binding of H1 resulted in a much more compact nucleosome structure with reduced distance between linker DNAs. The calculated linker DNA end-to-end distance decreased to ∼ 4.56 nm in the presence of H1. Similarly, EMSA assays show H1-bound nucleosomes migrate more rapidly through native PAGE than unbound WT nucleosomes, indicative of a more compact structure (Supplementary Figure S3). Surprisingly, we observed that despite the difference in linker DNA separation in WT and gH3 nucleosomes, linker DNA end-to-end distance was identical in H1-bound gH3 and WT nucleosomes (Figure 4A and B). These results suggest that a difference in linker DNA structure does not account for the effect of H3 tail deletion on H1 CTD structure.

**Figure 5.**
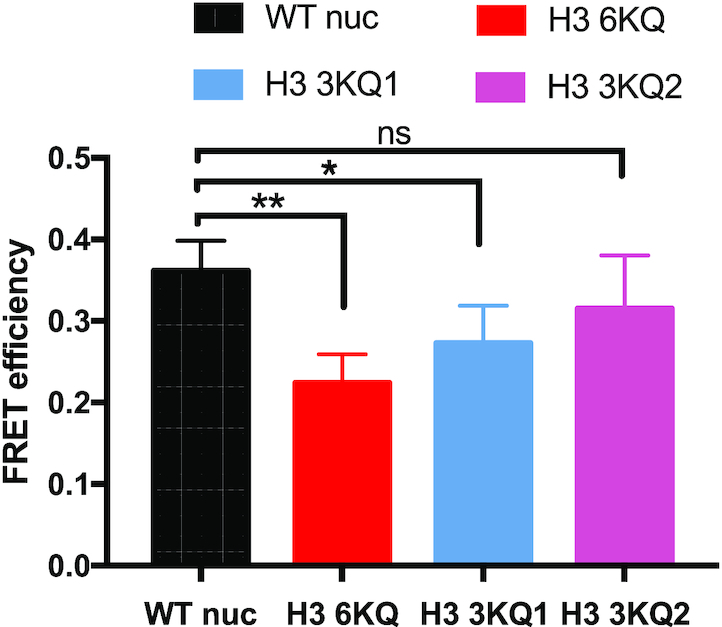
Linker DNA end-to-end distances in nucleosomes containing acetylation mimics do not correlate with effects on H1 CTD structure. FRET was used to estimate compare linker DNA end-to-end distances in WT, H3 6KQ, H3 3Q1 and H3 3KQ2 nucleosomes as in Figure 4. NS, no significant difference; (*) *P* < 0.05; (**) *P* < 0.01.

We next investigated linker DNA end-to-end distances in nucleosomes containing acetylation mimics. Similar to results for gH3 nucleosomes, H3 6KQ nucleosomes exhibited an opening of linker DNA ends compared to WT nucleosomes (Figure 5). Of note, inclusion of H3 3KQ1 also resulted in increased distance between linker DNA ends, although to a lesser extent compared to H3 6KQ, while no significant difference in end-to-end distance was found between H3 3KQ2 and WT nucleosomes. Notably, these results are in direct contrast to the effects of these mutations on H1 CTD structure, where we found that H3 3KQ2, but not H3 3KQ1, reduced H1 CTD condensation (Figure 3). Thus, the above data argues against the hypothesis that H3 N-terminal tail modifications affect H1 CTD condensation through altered linker DNA trajectory.

### Glutamine effectively models acetylated lysine

**Figure 6.**
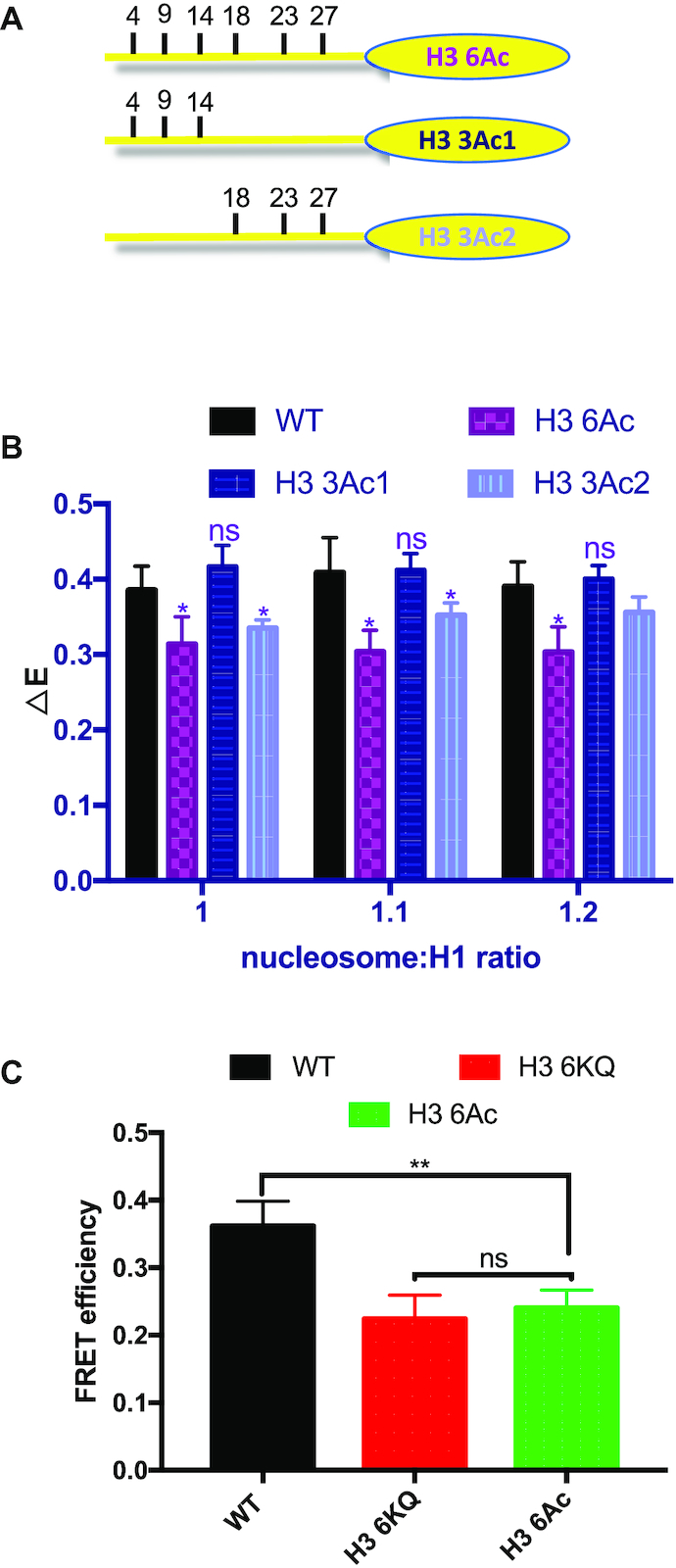
Lysine acetylation in the H3 tail domain affects H1 CTD condensation. H3 proteins containing acetylated lysines at known sites of lysine acetylation were incorporated into nucleosomes and the extent of H1 CTD condensation determined by FRET analysis. (**A**) Schematic showing sites of lysine acetylation in the H3 tail domain. (**B**) FRET analysis of labeled H1 incubated with WT and H3 6Ac, H3 3Ac1 and H3 3Ac2 nucleosomes at the indicated nucleosome: H1 ratios. NS, not significantly different from WT; (*) *P* < 0.05. Note calculated *P*-value for H3 3Ac2 at ratio 1.2 is 0.07 (not indicated). Analysis of aggregate data for samples in which H1-nuclesome binding is saturated (1.0, 1.1 and 1.2 ratios) indicates *P*-values of ≤ 3.0E–07, 0.30 and 6.7E-05 for H3 6Ac, H3 3Ac1 and H3 3Ac2, respectively, compared to WT (Supplementary Figure S6). (**C**) *Bona fide* lysine acetylation (6Ac) within H3 tail has identical effects on linker DNA end-to-end distance compared to K → Q acetylation mimic (6KQ). NS, not significantly different from WT; (**) *P* < 0.01.

Since the chemical structure of glutamine is not identical to acetylated lysine, we wished to determine whether the lysine to glutamine substitutions faithfully represented effects of *bona fide* acetylation on H1 CTD structure. We therefore prepared full-length histone H3 proteins by native chemical ligation that were homogeneously acetylated within the N-terminal tail. Three different acetylated proteins were generated: H3 6Kac, in which lysines K4, K9, K14, K18, K23 and K27 were acetylated, and H3 3Kac1 and H3 3Kac2, wherein lysines K4, K9 and K14, and K18, K23 and K27 were acetylated, respectively (Figure 6A, see also Supplementary Figure S5). These proteins were reconstituted with unmodified H2A, H2B and H4 to generate nucleosomes containing uniformly acetylated H3. Consistent with the previous experiments, H3 6Kac resulted in a significant decrease of H1 CTD condensation as indicated by a reduction in FRET compared to unmodified WT H3, similar to that observed with H3 6KQ (compare Figures 3B and 6B). Likewise, while the extent of H1 CTD condensation was not significantly different between nucleosomes containing WT H3 and H3 3Ac1, H3 3Ac2 elicited a reduction of H1 CTD condensation compared to unmodified WT nucleosomes, again paralleling the effects of the K→Q substitutions (Figure 6B). Finally, H3 6KAc nucleosomes exhibited a reduction (compared to WT nucleosomes) in the distance between linker DNA ends identical to that of H3 6KQ nucleosomes (Figure 6C) These analyses indicate that the K→Q acetylation mimics and *bona fide* acetylation within the H3 tail domain had virtually identical effects on H1 CTD condensation.

### Detection of interaction between the H3 N-terminal tail domain and H1 in the nucleosome

**Figure 7.**
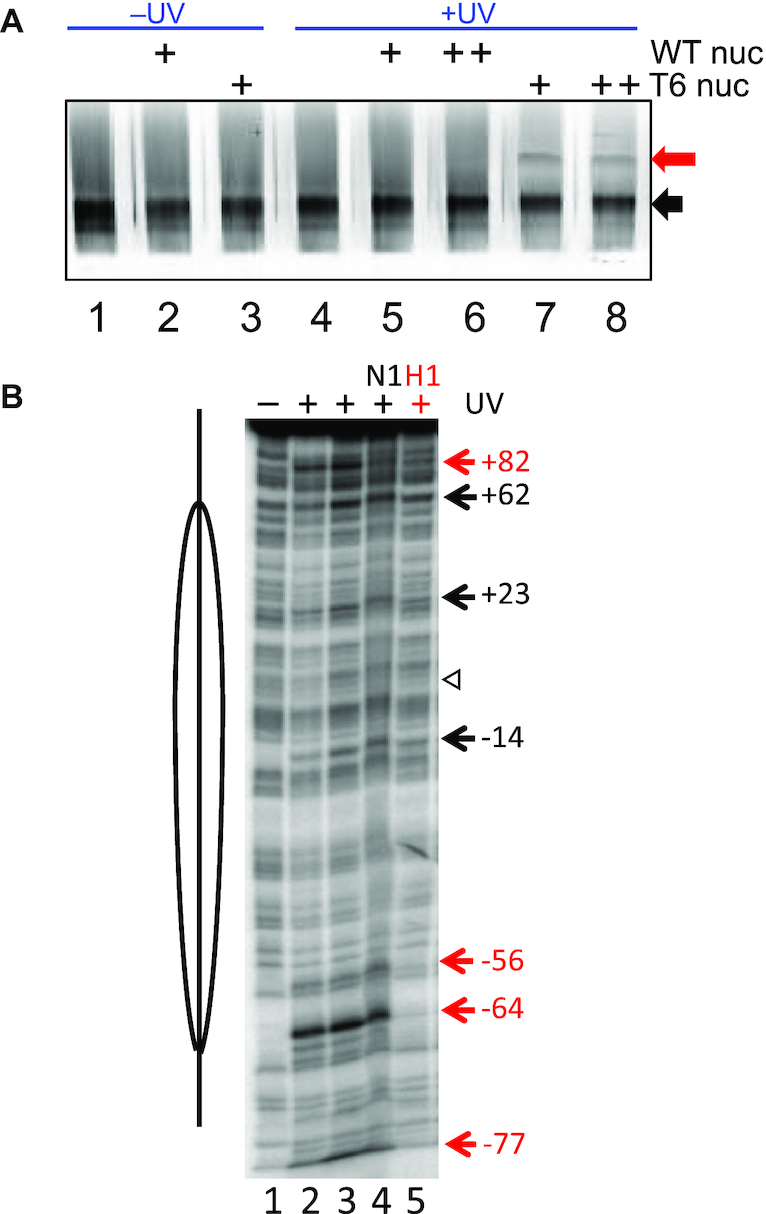
Evidence that the H3 tail domain physically interacts with the H1 CTD. (**A**) The H3 tail domain crosslinks to H1 in nucleosomes. Cy5-labeled H1was incubated with nucleosomes containing APB-modified H3 T6C, crosslinking initiated with UV light, and products separated on SDS-PAGE gels. Gels were imaged by fluorography detecting Cy5. The black arrow indicates the position of uncrosslinked H1, while the red arrow indicates the position of a presumed H3 T6C-APB-H1 crosslinking product. (**B**) H1 disrupts specific H3 tail-DNA crosslinking sites in nucleosomes. Nucleosomes reconstituted with H3 T6C-APB and 5′ end-radiolabeled DNA were irradiated and crosslinks visualized as strand breaks on 5% DNA denaturing gels (37). Lane 1, no irradiation, lanes 2–5, irradiated samples. Lanes 1–3 contain nucleosomes alone, while lanes 4 and 5 contain nucleosomes incubated with HMGN1 or H1, respectively. The location of the nucleosome dyad is indicated by the open arrowhead. Numbers indicate sites of crosslinks unaffected (black) or affected (red) by H1 binding.

We next asked whether the H3 N-terminal tail domain exists in close enough proximity to H1 to allow protein-protein crosslinking within the nucleosome. We employed a site-specific crosslinking approach in which a histone residue is modified with azidophenacyl bromide (APB), and crosslinking initiated by a brief irradiation with UV light. Crosslinking occurs to either protein or DNA within 0–12Å of the Cα carbon of the APB-modified cysteine (37,47). To determine if the H3 tail comes in close proximity to H1 in the nucleosome, we modified the H3 mutant H3 T6C with APB and incorporated the modified protein into nucleosomes (37). We bound Cy5-labeled H1 G101C to nucleosomes and activated crosslinking via a brief UV irradiation. The H1-nucleosome complexes were then loaded on SDS-PAGE gels and analyzed by fluorography to allow the detection of fluorescently labeled H1 and any species covalently crosslinked to H1. No self-crosslinking of H1 is observed upon UV irradiation (Figure 7A, compare lane 1 and 4). In the absence of UV irradiation, only one band representing Cy5 labeled H1 was detected on the gel. However, upon UV irradiation, a novel band appeared on the gel above H1(Figure 7A, lanes 7 and 8). In addition, the new band is dependent on H3T6C APB modification, indicating the novel band is the product of UV-induced crosslinking between the N-terminal tail domain of H3 and H1 (Figure 7A, compare lanes 5, 6 and 7, 8). These data suggest H3 N-terminal tail contacts H1 within the mononucleosome.

To further investigate H3 tail interactions in the nucleosome, we examined crosslinking of H3 T6C-APB to nucleosome DNA in the absence and presence of H1. In previous work we found that APB modified H3 T6C crosslinks to nucleosome DNA at +82, +62, +23, –14, –56, –64 and –77 nt from the dyad (defined as position 0), forming two clusters of interactions on the nucleosome DNA surface (37). One cluster is located more internally within the nucleosome (composed of +62, +23, –14, –56 and –64) while a second cluster is located in the linker DNA region (composed of +82 and –77). Our current crosslink mapping data is entirely consistent with these findings (Figure 7B, compare lanes 1 and 3). In the previous work, also found that the architectural chromatin factor HMGN1 selectively disrupted specific H3 tail-DNA interactions, consistent with current results (lane 4). Importantly, we find that binding of H1 to the nucleosome diminishes crosslinking at the linker DNA sites (–77, +82) and sites near the nucleosome core periphery (–56, –64) but not at more internal sites (Figure 7B, lane 5). Taken together our crosslinking results suggest that binding of H1 redirects interactions of end of the H3 tail domain away from the linker DNA, possibly by direct contact with the H1 CTD in the nucleosome.

## DISCUSSION

We present evidence for a novel communication between the H3 tail domain and the H1 CTD. Our data indicate that the H3 tail domain directly influences H1 CTD structure and that specific acetylation events in the H3 tail alter H1 CTD structure. We find that acetylation or modifications at other positions within the H3 tail or of other N-terminal tail domains within the nucleosome do not significantly influence CTD structure. Finally, we provide evidence that the H3 tail directly interacts with the H1 CTD to regulate its structure. Interestingly, acetylation at position K18, K23 and K27 of the H3 N-terminal tail, but not those near the most N-terminus (K4, K9 and K14), result in a reduction of H1 CTD condensation, suggesting specific acetylation events cause distinct changes in H3 tail conformations within the nucleosomal context (48). Acetylation removes the positive charge on the lysine side-chain. It has been shown that H3 tail robustly interacts with both nucleosomal and linker DNA (37,48). Thus, the more interior acetylations along the H3 tail might result in an altered conformation adopted by the bound tail.

Our previous work indicates the intrinsically disordered H1 CTD undergoes significant condensation upon binding to nucleosomes that is consistent with adoption of a defined structure or ensemble of structures. Evidence suggests that the entropic cost associated with CTD condensation appears to be offset by the significant positive contributions to overall binding free energy derived from interaction of the ∼40 excess positive charges within the CTD with DNA (25). Indeed, the H1 CTD is essential for stabilization of higher order chromatin structures due to neutralizing charge within linker DNA, suggesting H1 CTD structure plays a critical role in this function. Importantly, H1 CTD structure is coupled to at least an initial step in the folding of extended oligonucleosome arrays into higher order structures (6), indicating that the propensity for the H1 CTD to adopt various condensed states likely influences the stability of chromatin folding. Given that acetylation within the H3 tail directly alters H1 CTD structure, our work therefore identifies a potentially new mechanism by which acetylation influences higher order chromatin structure.

Despite the presumed role of H1 as a general transcriptional repressor, a 50% reduction of H1 expression in mouse embryonic stem (ES) cells resulted in remarkably few significant changes in the expression of specific genes (49). However, H1 was found to direct epigenetic regulation of the imprinting genes H19 and Gtl2 through two different mechanisms. First, H1 physically interacts with DNA methyltransferase DNMT3B and DNMT1 to promote the establishment and maintenance of DNA methylation that leads to gene repression at these loci; second, H1 can hinder the binding of SET7/9 and H3K4 methylation, a modification associated with transcriptional activation (50). Of note, the H1 CTD region is required for the direct interaction between H1 and the DNA methyltransferases. Additionally, it has been reported that a direct interaction between H1 and heterochromatin-specific H3 K9 methyltransferase Su(var)3–9 is essential for the repression of transposable elements in Drosophila heterochromatin (51). Importantly, partial or full-length H1 CTD deletion results in strong activation of the transposable elements similar to that observed above for H1 knockdown (52). Such interactions may depend on the structure of the H1 CTD, and thus in turn may be regulated by modifications within the H3 tail domain.

We previously showed that two members of the HMGN family of architectural transcription factors, HMGN 1 and HMGN2 alter H1 CTD condensation while not disturbing H1 globular domain binding at the nucleosome dyad (37). Moreover, it has been shown that phosphorylation within peptides derived from H1 CTD domains alters the propensity for folding/condensation in solvents and salts that model the chemical environment in chromatin (53), and also in native chromatin (54,55). Taken with our current findings, these data suggest that the H1 CTD may be a nexus for signaling in the nucleosome. In this regard, understanding H1 CTD structure and factors that alter the final state of this intrinsically disordered domain in chromatin will be critical for deciphering the regulation of chromatin states.

## Supplementary Material

gkaa949_Supplemental_File
