# Supplementary material for: Acetylation-modulated communication between the H3 N-terminal tail domain and the intrinsically disordered H1 C-terminal domain
Source: Nucleic Acids Res. 2020 Oct 30;48(20):11510–20. doi: 10.1093/nar/gkaa949 (PMC7672455; doi:10.1093/nar/gkaa949)
Supplement: gkaa949_Supplemental_File [file gkaa949_supplemental_file.pptx]

## Slide 1
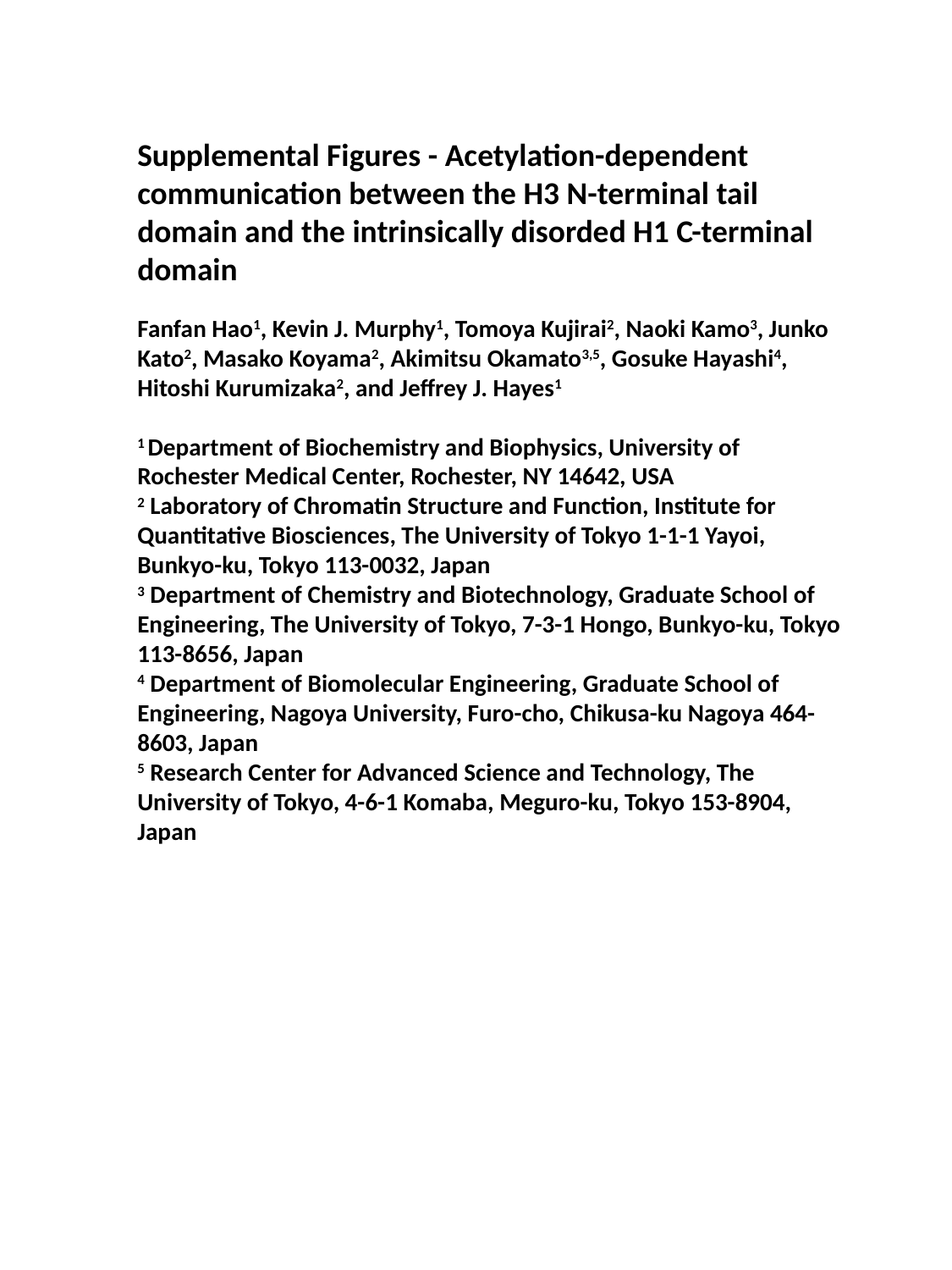

Supplemental Figures - Acetylation-dependent communication between the H3 N-terminal tail domain and the intrinsically disorded H1 C-terminal domain
Fanfan Hao1, Kevin J. Murphy1, Tomoya Kujirai2, Naoki Kamo3, Junko Kato2, Masako Koyama2, Akimitsu Okamato3,5, Gosuke Hayashi4, Hitoshi Kurumizaka2, and Jeffrey J. Hayes1
1 Department of Biochemistry and Biophysics, University of Rochester Medical Center, Rochester, NY 14642, USA
2 Laboratory of Chromatin Structure and Function, Institute for Quantitative Biosciences, The University of Tokyo 1-1-1 Yayoi, Bunkyo-ku, Tokyo 113-0032, Japan
3 Department of Chemistry and Biotechnology, Graduate School of Engineering, The University of Tokyo, 7-3-1 Hongo, Bunkyo-ku, Tokyo 113-8656, Japan
4 Department of Biomolecular Engineering, Graduate School of Engineering, Nagoya University, Furo-cho, Chikusa-ku Nagoya 464-8603, Japan
5 Research Center for Advanced Science and Technology, The University of Tokyo, 4-6-1 Komaba, Meguro-ku, Tokyo 153-8904, Japan

## Slide 2
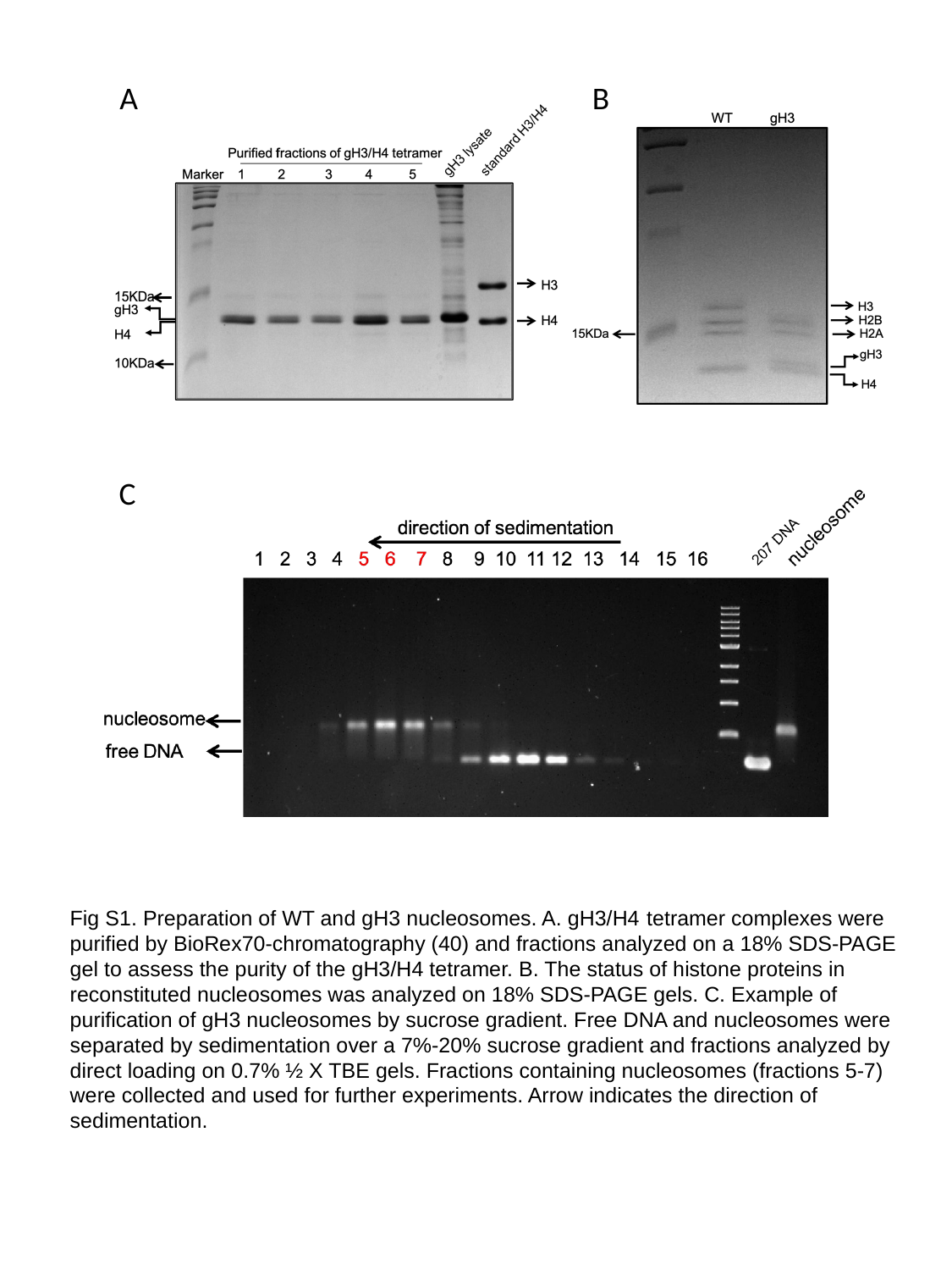

A
B
C
Fig S1. Preparation of WT and gH3 nucleosomes. A. gH3/H4 tetramer complexes were purified by BioRex70-chromatography (40) and fractions analyzed on a 18% SDS-PAGE gel to assess the purity of the gH3/H4 tetramer. B. The status of histone proteins in reconstituted nucleosomes was analyzed on 18% SDS-PAGE gels. C. Example of purification of gH3 nucleosomes by sucrose gradient. Free DNA and nucleosomes were separated by sedimentation over a 7%-20% sucrose gradient and fractions analyzed by direct loading on 0.7% ½ X TBE gels. Fractions containing nucleosomes (fractions 5-7) were collected and used for further experiments. Arrow indicates the direction of sedimentation.

## Slide 3
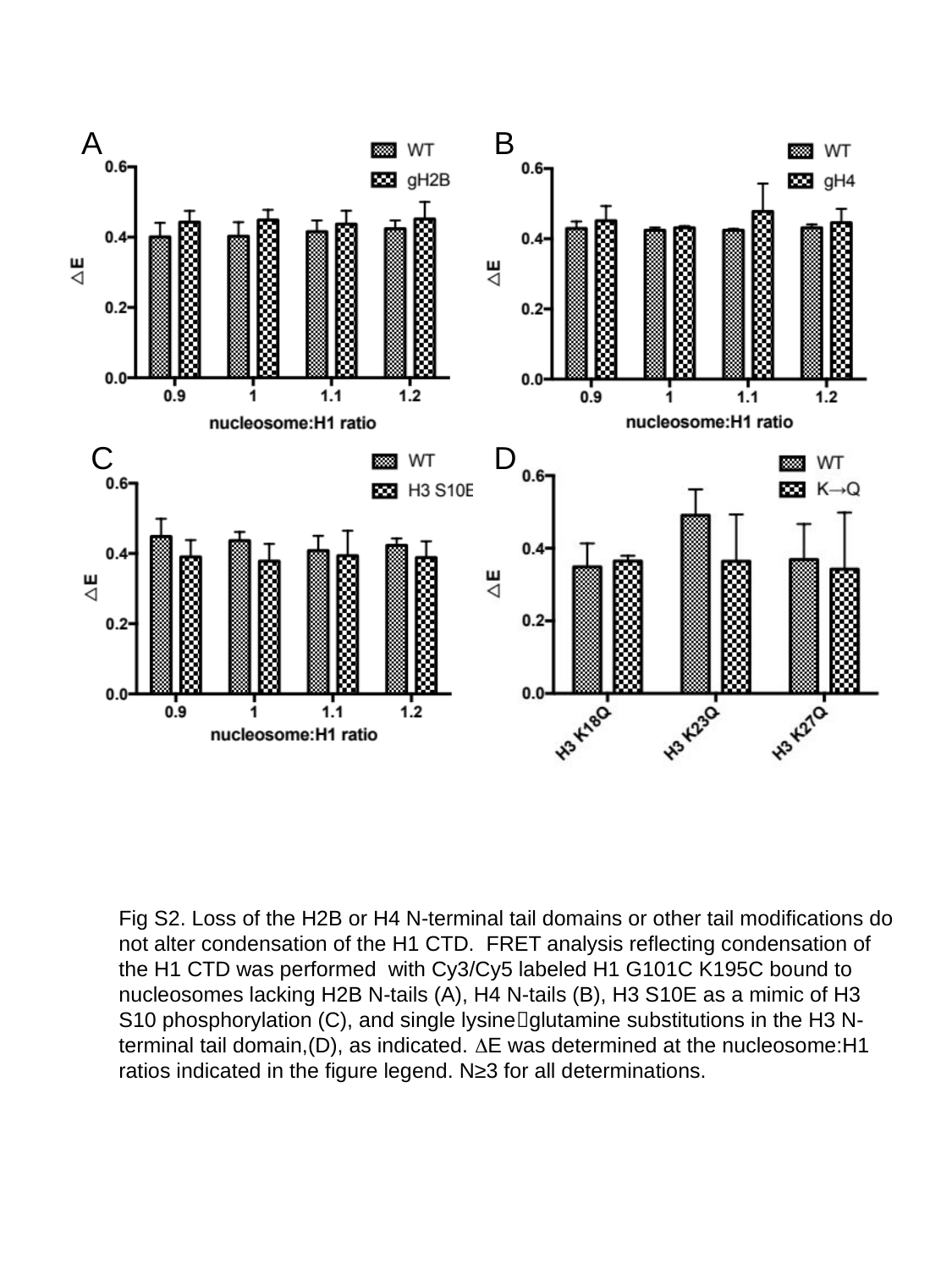

A
B
C
D
Fig S2. Loss of the H2B or H4 N-terminal tail domains or other tail modifications do not alter condensation of the H1 CTD. FRET analysis reflecting condensation of the H1 CTD was performed with Cy3/Cy5 labeled H1 G101C K195C bound to nucleosomes lacking H2B N-tails (A), H4 N-tails (B), H3 S10E as a mimic of H3 S10 phosphorylation (C), and single lysineglutamine substitutions in the H3 N-terminal tail domain,(D), as indicated. DE was determined at the nucleosome:H1 ratios indicated in the figure legend. N≥3 for all determinations.

## Slide 4
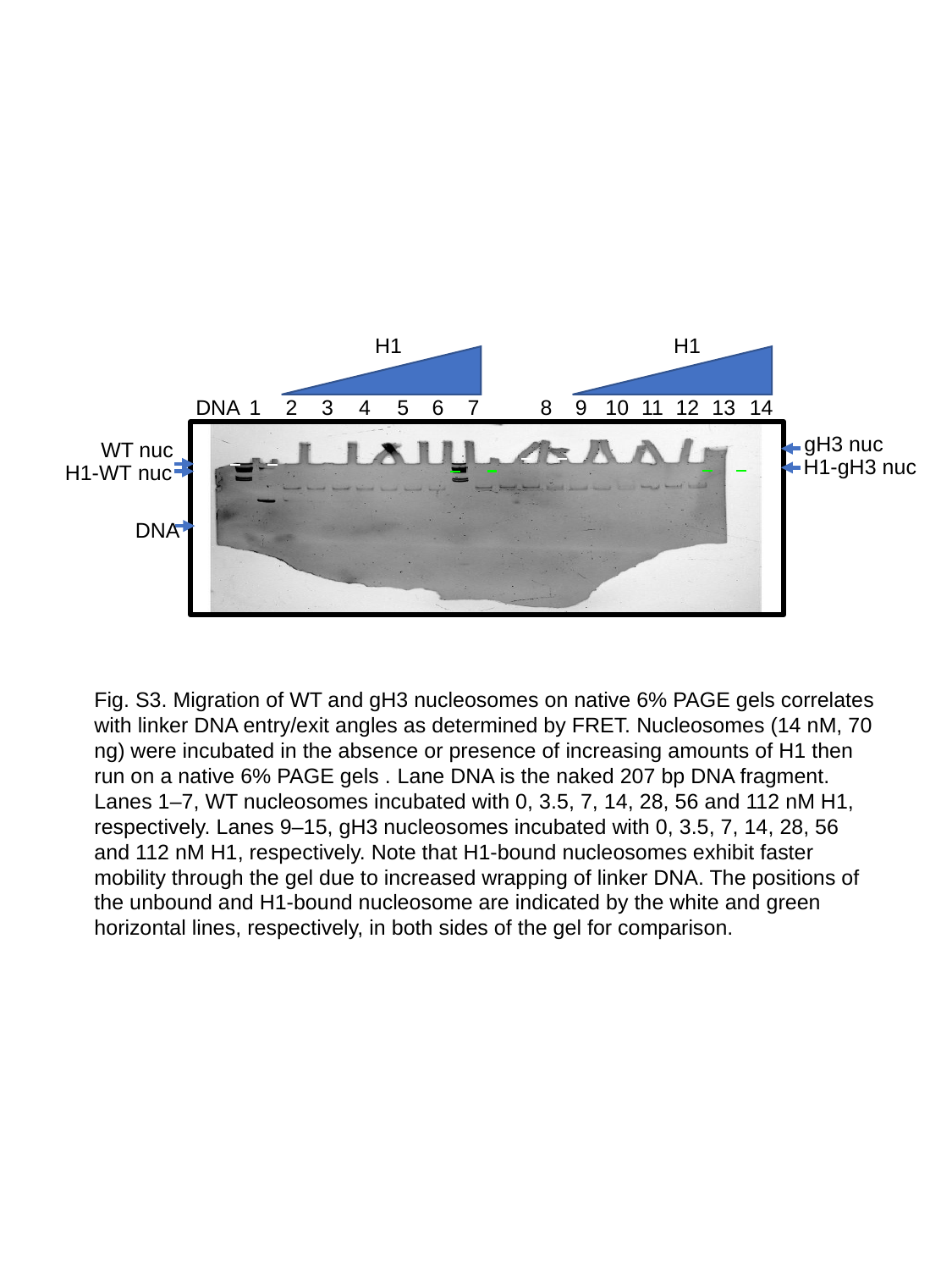

H1
H1
DNA
1
2
3
4
5
6
7
8
9
10
11
12
13
14
gH3 nuc
 WT nuc
H1-gH3 nuc
H1-WT nuc
DNA
Fig. S3. Migration of WT and gH3 nucleosomes on native 6% PAGE gels correlates with linker DNA entry/exit angles as determined by FRET. Nucleosomes (14 nM, 70 ng) were incubated in the absence or presence of increasing amounts of H1 then run on a native 6% PAGE gels . Lane DNA is the naked 207 bp DNA fragment. Lanes 1–7, WT nucleosomes incubated with 0, 3.5, 7, 14, 28, 56 and 112 nM H1, respectively. Lanes 9–15, gH3 nucleosomes incubated with 0, 3.5, 7, 14, 28, 56 and 112 nM H1, respectively. Note that H1-bound nucleosomes exhibit faster mobility through the gel due to increased wrapping of linker DNA. The positions of the unbound and H1-bound nucleosome are indicated by the white and green horizontal lines, respectively, in both sides of the gel for comparison.

## Slide 5
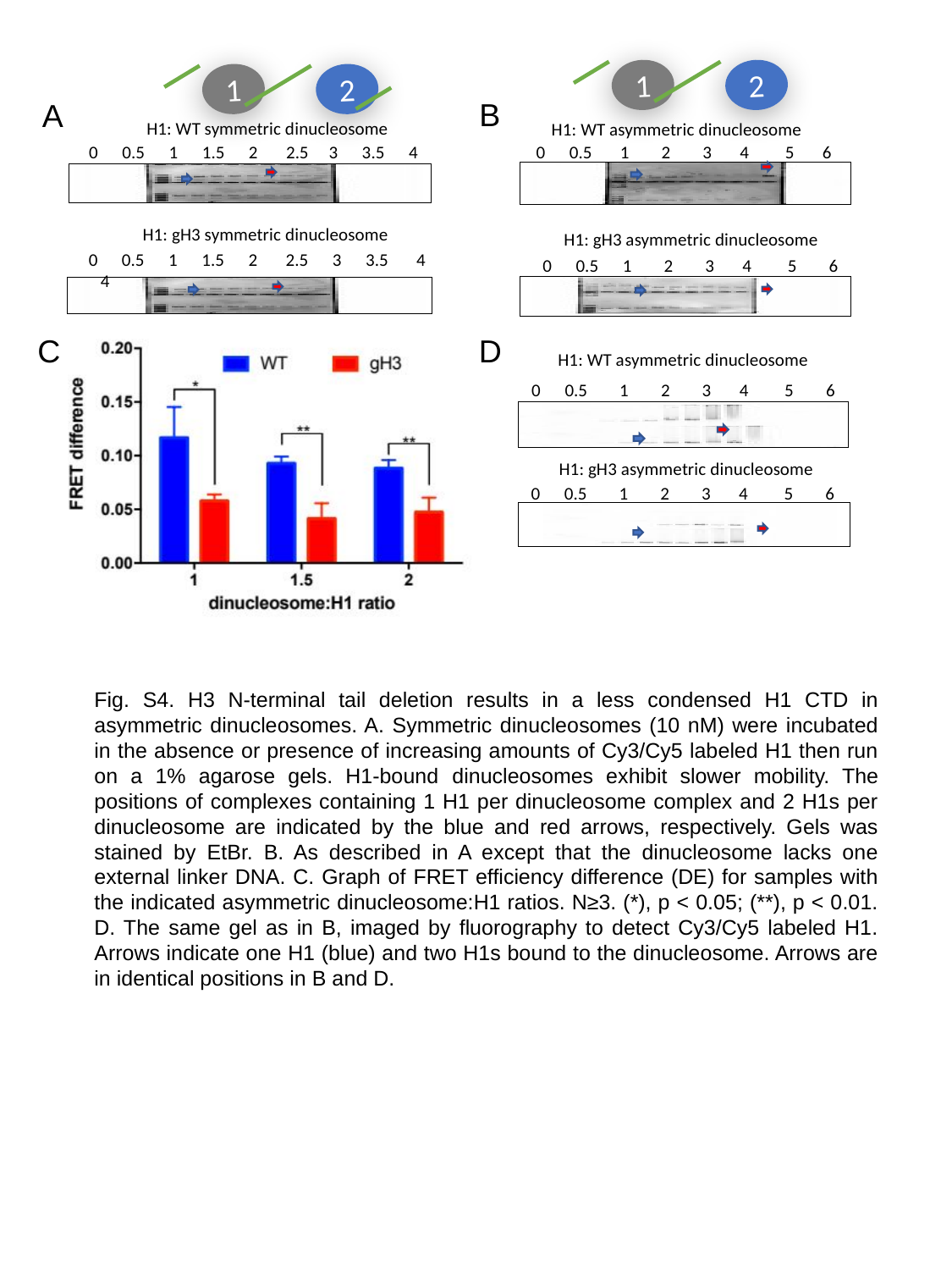

1
2
1
2
B
A
H1: WT symmetric dinucleosome
0 0.5 1 1.5 2 2.5 3 3.5 4
H1: WT asymmetric dinucleosome
0 0.5 1 2 3 4 5 6
H1: gH3 symmetric dinucleosome
H1: gH3 asymmetric dinucleosome
0 0.5 1 2 3 4 5 6
0 0.5 1 1.5 2 2.5 3 3.5 4 4
C
D
H1: WT asymmetric dinucleosome
0 0.5 1 2 3 4 5 6
H1: gH3 asymmetric dinucleosome
0 0.5 1 2 3 4 5 6
Fig. S4. H3 N-terminal tail deletion results in a less condensed H1 CTD in asymmetric dinucleosomes. A. Symmetric dinucleosomes (10 nM) were incubated in the absence or presence of increasing amounts of Cy3/Cy5 labeled H1 then run on a 1% agarose gels. H1-bound dinucleosomes exhibit slower mobility. The positions of complexes containing 1 H1 per dinucleosome complex and 2 H1s per dinucleosome are indicated by the blue and red arrows, respectively. Gels was stained by EtBr. B. As described in A except that the dinucleosome lacks one external linker DNA. C. Graph of FRET efficiency difference (DE) for samples with the indicated asymmetric dinucleosome:H1 ratios. N≥3. (*), p < 0.05; (**), p < 0.01. D. The same gel as in B, imaged by fluorography to detect Cy3/Cy5 labeled H1. Arrows indicate one H1 (blue) and two H1s bound to the dinucleosome. Arrows are in identical positions in B and D.

## Slide 6
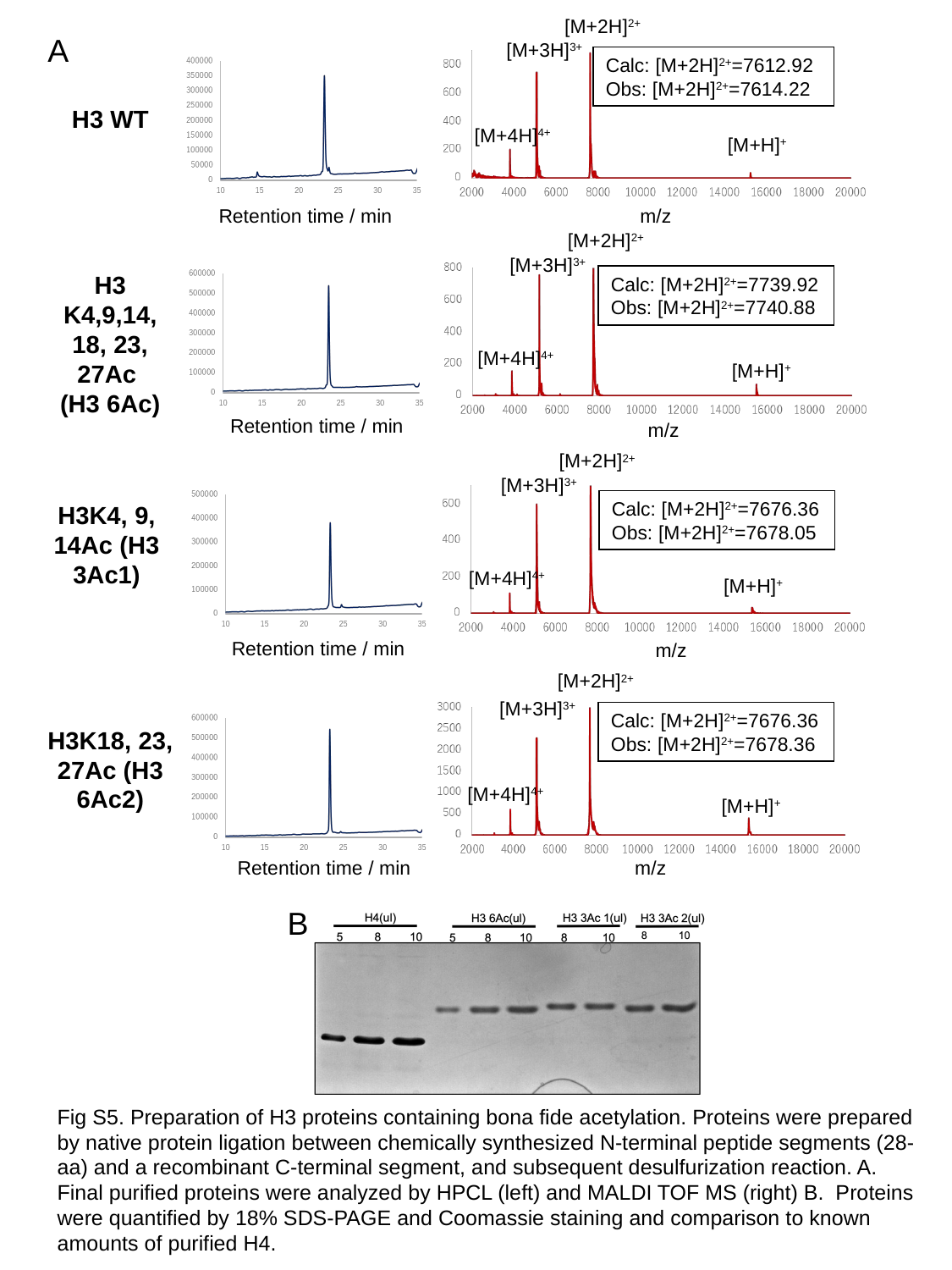

[M+2H]2+
[M+3H]3+
Calc: [M+2H]2+=7612.92
Obs: [M+2H]2+=7614.22
[M+H]+
m/z
H3 WT
[M+4H]4+
Retention time / min
A
[M+2H]2+
[M+3H]3+
[M+4H]4+
[M+H]+
m/z
H3 K4,9,14, 18, 23, 27Ac
(H3 6Ac)
Retention time / min
Calc: [M+2H]2+=7739.92
Obs: [M+2H]2+=7740.88
[M+2H]2+
[M+3H]3+
[M+H]+
m/z
H3K4, 9, 14Ac (H3 3Ac1)
[M+4H]4+
Retention time / min
Calc: [M+2H]2+=7676.36
Obs: [M+2H]2+=7678.05
[M+2H]2+
[M+3H]3+
[M+H]+
m/z
H3K18, 23, 27Ac (H3 6Ac2)
[M+4H]4+
Retention time / min
Calc: [M+2H]2+=7676.36
Obs: [M+2H]2+=7678.36
B
Fig S5. Preparation of H3 proteins containing bona fide acetylation. Proteins were prepared by native protein ligation between chemically synthesized N-terminal peptide segments (28-aa) and a recombinant C-terminal segment, and subsequent desulfurization reaction. A. Final purified proteins were analyzed by HPCL (left) and MALDI TOF MS (right) B. Proteins were quantified by 18% SDS-PAGE and Coomassie staining and comparison to known amounts of purified H4.

## Slide 7
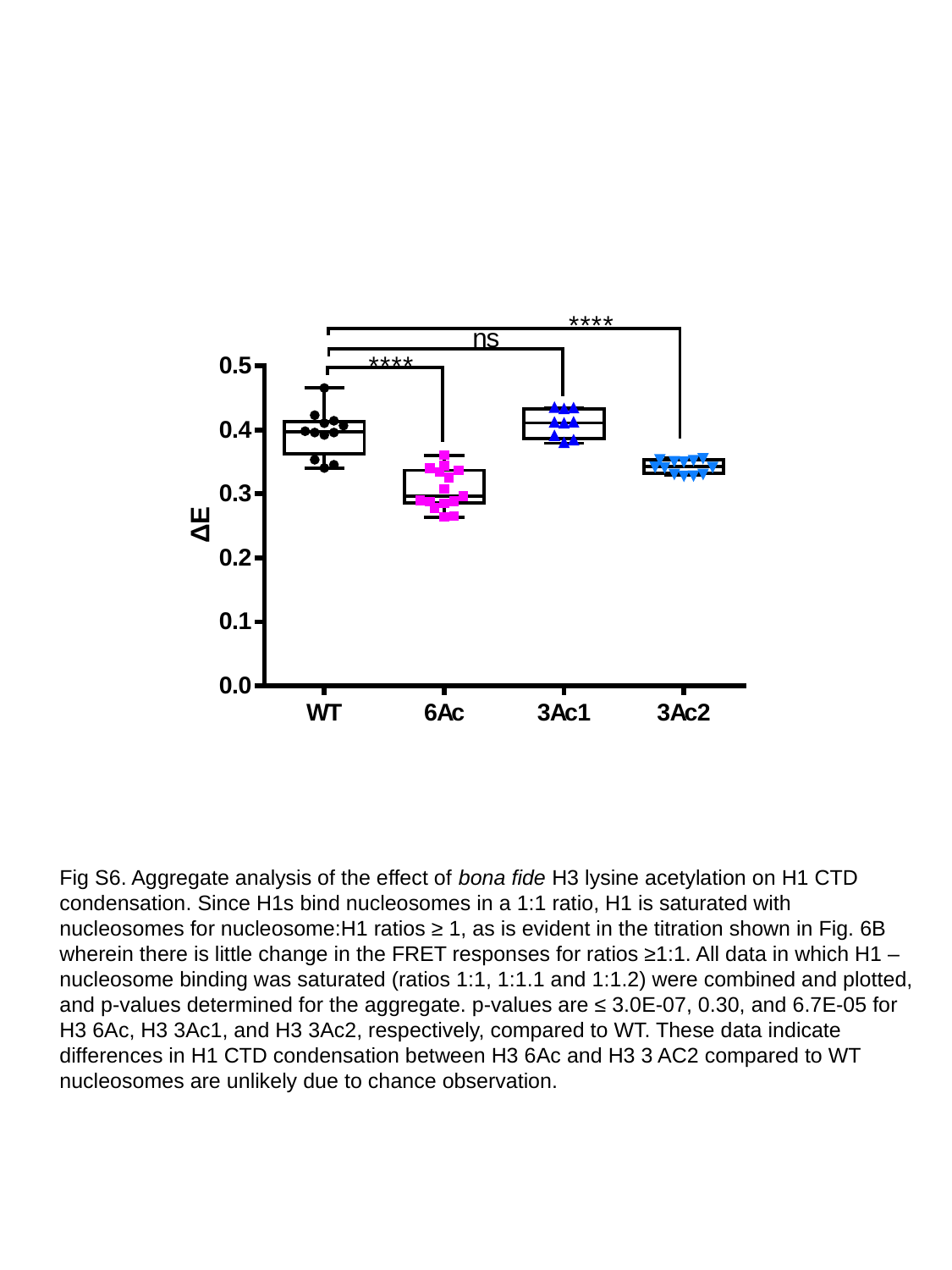

Fig S6. Aggregate analysis of the effect of bona fide H3 lysine acetylation on H1 CTD condensation. Since H1s bind nucleosomes in a 1:1 ratio, H1 is saturated with nucleosomes for nucleosome:H1 ratios ≥ 1, as is evident in the titration shown in Fig. 6B wherein there is little change in the FRET responses for ratios ≥1:1. All data in which H1 – nucleosome binding was saturated (ratios 1:1, 1:1.1 and 1:1.2) were combined and plotted, and p-values determined for the aggregate. p-values are ≤ 3.0E-07, 0.30, and 6.7E-05 for H3 6Ac, H3 3Ac1, and H3 3Ac2, respectively, compared to WT. These data indicate differences in H1 CTD condensation between H3 6Ac and H3 3 AC2 compared to WT nucleosomes are unlikely due to chance observation.
